# The Association between SARS-CoV-2 Infection and Diabetic Ketoacidosis in Patients with New-Onset Diabetes: A Retrospective Study from a Diabetic Center in Saudi Arabia

**DOI:** 10.3390/pediatric14040060

**Published:** 2022-11-29

**Authors:** Eman Hurissi, Mohammed Alameer, Fadiyah Ageeli, Maram Allami, Mohammed Alharbi, Hussam Suhail, Hadeel Albeishy, Omar Oraibi, Mohammed Somaili, Abdulrahman Hummadi, Abdulaziz H. Alhazmi

**Affiliations:** 1 Faculty of Medicine, Jazan University, Jazan 45142, Saudi Arabia; 2 Jazan Endocrinology and Diabetes Center, Ministry of Health, Jazan 82722, Saudi Arabia

**Keywords:** COVID-19, diabetes, DKA, DM, Jazan, Saudi Arabia

## Abstract

Background: Various reports described new-onset diabetes during or after severe acute respiratory syndrome coronavirus 2 (SARS-CoV-2) infection in patients with no previous history of diabetes or glucocorticoid use. Further, SARS-CoV-2 could increase the risk of diabetes, including diabetic ketoacidosis (DKA). However, data on the relationship between new-onset diabetes and COVID-19 are still limited in our region. Thus, we aimed in this study to evaluate the association between new-onset diabetes and DKA in patients with COVID-19. Methods: A retrospective, cross-sectional study was conducted at a diabetic center in Jazan province, Saudi Arabia, between 2020 and 2021. Demographic data, COVID-19 status, and DKA incidence were collected and verified manually from diabetic patients’ medical records. Data were analyzed using a t-test and chi-square test. Results: We included 54 diabetic patients diagnosed during the COVID-19 pandemic, with a median age of 17 years. The majority of patients were females (57.4%). About 38.8% were diagnosed with COVID-19, and 16.6% reported having DKA. About 33.3% of the patients who experienced DKA reported being COVID-19-positive. However, only 6% of patients who denied contracting SARS-CoV-2 developed DKA (*p*-value = 0.020). Conclusions: Patients with newly diagnosed diabetes due to COVID-19 seem at a higher risk of developing DKA. Further epidemiological and molecular studies are required for a better understanding of the correlation between DKA in patients with diabetes and COVID-19.

## 1. Introduction

In December 2019, a cluster of pneumonia cases of unknown cause emerged from the seafood wholesale market in Wuhan city, China [1]. This infection has rapidly spread and become a global public threat. A novel coronavirus was isolated from human epithelial cells and named 2019-nCoV; after genome sequencing and phylogenetic analysis, the findings showed that 2019-nCoV belongs to the same family as severe acute respiratory syndrome coronavirus 1 (SARS-CoV-1) and Middle East respiratory syndrome coronavirus (MERS-CoV), and it was therefore named SARS-CoV-2 [1]. After documenting over 118,000 cases worldwide in 114 countries, the World Health Organization (WHO) declared coronavirus disease 2019 (COVID-19) as a pandemic on 11 March 2020 [2]. Like all coronaviruses, SARS-CoV-2 is a single-stranded RNA virus that could evolve and induce genetic mutations over time while being transmitted from one host to another [3].

The first clinical description for SARS-CoV-2 infection found that the common symptoms were fever (98.6%), fatigue (69.6%), and dry cough (59.4%), with 26.1% of patients being shifted to the intensive care unit (ICU). The complications of SARS-CoV-2 infection include acute respiratory distress syndrome (ARDS), shock, and arrhythmia. Compared to patients not admitted to the ICU, patients in the ICU were older and more likely to have underlying comorbidities such as diabetes mellitus (DM) [4]. Previously, DM was recognized as an independent factor associated with poor clinical outcomes in patients infected with SARS-CoV-1 [5] and MERS-CoV [6]. Furthermore, newly diagnosed diabetes was commonly detected during SARS-CoV-1 outbreaks in patients with no previous history of DM or glucocorticoid use [5]. A study by Yang et al. aimed to determine whether the multiple organ damage in SARS-CoV-1 patients was related to different organ expressions of the coronaviruses receptor, angiotensin-converting enzyme 2 (ACE2), including endocrine tissues in the pancreas. They highlighted that the damage to islets was transient, and the mechanism of newly diagnosed diabetes is due to the localization of ACE2 expression in the endocrine part of the pancreas [7].

DM may also be associated with severe COVID-19 [8]. New-onset hyperglycemia is considered one of the complications of COVID-19, primarily among hospitalized patients. Interestingly, new-onset hyperglycemia is not correlated with other risk factors, such as prediabetes, obesity, previous DM, or corticosteroid use [9]. There are multiple studies and case series that reported an association between COVID-19 and the exacerbation of high blood glucose condition, which could be manifested by diabetic ketoacidosis (DKA) [10,11]. For example, a multicenter retrospective cohort study conducted by Alaqeel et al. found that new-onset diabetes and DKA were higher in 2020 than in 2019 in Saudi Arabia [12].

Thus, in this study, we aimed to describe our experience in a diabetic center in southwestern Saudi Arabia that serves about 2 million people and to identify the association between new-onset diabetes and DKA in relation to SARS-CoV-2 infection.

## 2. Materials and Methods

### 2.1. Study Design and Participants

A retrospective cross-sectional study was conducted at the diabetic center in Jazan province, Saudi Arabia. Demographic data, COVID-19 infection status, and DKA incidence were collected manually between 1 December 2021 and 1 July 2022 from patients’ medical records and verified by contacting patients or their guardians.

### 2.2. Target Population

The target population was patients with newly diagnosed diabetes who visited the diabetic center and were diagnosed with diabetes during the COVID-19 pandemic (i.e., between 2020 and 2021). We assumed that our participants had type 1 diabetes (T1D) considering their younger age and BMI, as the majority of them were underweight (44.44%). Patients with COVID-19 were those with a positive SARS-CoV-2 nasopharyngeal test. DKA was defined as an emergency condition resulting from high blood sugar that required the affected individual to visit an emergency room and receive the required treatment, and further confirmed via hyperglycemia, ketonuria, and acidosis. These data were retrieved from patients’ medical records and verified by contacting the patients or their guardians. The study excluded data from participants who provided incomplete responses and responses that did not contain informed consent.

### 2.3. Sample Size Calulcation

The incidence rate of T1D in Saudi Arabia is about 35 per 100,000 individuals, with a steadily rising incidence of 3% per year [13]. Thus, the sample size was calculated based on these data, and we assumed that the number of patients diagnosed with T1D in Jazan was about 1400 patients, with an incidence of 42 patients per year.

### 2.4. Statistical Analysis

A statistical data entry and analysis were performed using SPSS v.23 (IBM Corp.: Armonk, NY, USA). Data analyses involved descriptive statistics as well as inferential statistics, according to the required purpose of each relationship. Normally, distribution data were managed by tests that were appropriate for this type of data, e.g., a *t*-test and ANOVA test. All categorical variables were presented as frequencies and percentages, while continuous variables were presented as means and standard deviations. The association between two categorical variables was investigated using the chi-square test. Statistical significance was set at a *p*-value <0.05.

### 2.5. Ethical Approval

The study was approved by Jazan Health Ethics Committee, Ministry of Health, Saudi Arabia, with approval number #2171 (dated 30 September 2021). This study was conducted following the ethical guidelines of the Helsinki Declaration and the local guidelines of the National Committee of Bioethics, Saudi Arabia. Data had been collected for clinical purposes and were available on medical record databases. Collected data were kept confidential and only used for the purpose of research within the objectives of this research. Additionally, we did not include participants’ data or any other methods of identification.

## 3. Results

Our final sample consisted of 54 participants. The participants’ age ranged from 10 to 23 years old, with a mean of 17 years and a standard deviation of 3. About 42.5% of participants were male, while 57.4% were females. According to the Body Mass Index (BMI), 44.4% were underweight, 42.5% were normal, 11.1% were overweight, and 1.8% were obese. About 38% of participants were COVID-19-positive (i.e., the SARS-CoV-2 nasopharyngeal test was positive in their medical record), and out of these patients, 16.6% had DKA as a complication of COVID-19. These results are summarized in Table 1.

**Figure 1:**
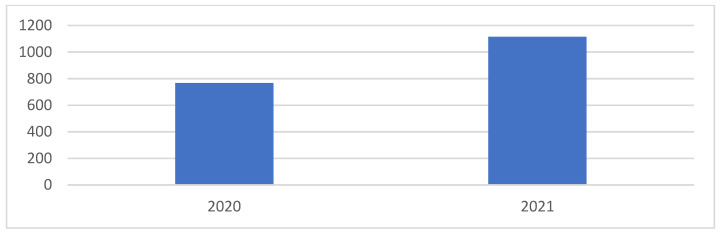
Prevalence of newly diagnosed patients with DM during the COVID-19 pandemic (*p*-value = 0.0001).

Figure 1 shows the prevalence of newly diagnosed patients with diabetes before and during the COVID-19 pandemic, according to the diabetic center in the Jazan region. The prevalence of newly diagnosed patients with diabetes in 2020 was 768, and in 2021 it was 1115 (*p*-value = 0.0001).

Then, we compared patients with COVID-19 against those who were negative for COVID-19. The proportion of DKA among patients with COVID-19 was 33%, and the proportion of DKA among patients without COVID-19 was 6% (*p*-value = 0.020). Further analyses are summarized in Table 2.

## 4. Discussion

New-onset diabetes has been repeatedly linked to some viral infections [14,15]. It has been suggested that the cellular receptors are crucial factors in the association between diabetes and coronavirus infections including SARS, MERS, and COVID-19 [16,17]. Moreover, SARS-CoV-2 is considered a potential risk factor for having DKA in patients with diabetes. Our study found an association between COVID-19 and the development of DKA in patients with newly diagnosed diabetes. The results of our study showed that 33% of our patients with diabetes who had contracted SARS-CoV-2 developed DKA compared to 6% of patients with negative COVID-19 (Table 2), and these results are consistent with many studies that reported an association between new-onset diabetes with DKA and SARS-CoV-2 infection (Table 3).

A study in the UK showed that a total of 30 children presented with new-onset T1D during the pre-pandemic period compared to 17 children during the first COVID-19 wave. Children presented more frequently with DKA during the first COVID-19 wave in comparison with the pre-pandemic period. During the first COVID-19 wave, the results showed that the children frequently presented with a high percentage of severe DKA (47%) and higher hemoglobin A1c (HbA1c), a test that measures the average level of blood sugar over the past 2 to 3 months. DKA presentations in children with a family history of T1D were less severe in comparison to those without a family history. The author stated that the COVID-19 pandemic was associated with an increased severity of childhood-onset T1D [18]. A similar study was conducted in Germany on 532 children and adolescents with newly diagnosed T1D between 13 March and 13 May 2020. DKA was present in 238 patients (44.7%), and severe DKA was present in 103 patients (19.4%). During the COVID-19 period in 2020, the frequency of DKA was significantly higher compared with previous years. The incidence of severe DKA was also significantly higher compared with the previous year [19]. In a study conducted in Israel, the results showed that during the COVID-19 period, the DKA incidence was 58.2% and was significantly higher than in 2019. During the COVID-19 pandemic, children aged 6–11 years had higher incidences of DKA [20]. Another study conducted in the UK from Northwest London, regarding new-onset T1D and DKA during the peak of the COVID-19 pandemic between 23rd March and 4th June 2020, showed an increase in DKA cases of up to 70%, with up to 52% presenting with severe DKA [21]. The local figure in Saudi Arabia did not seem different from what was previously reported. A study was conducted on 106 children who were admitted to diabetic centers during lockdown in 2020 compared with 154 in 2019. Among admitted children, DKA was higher in 2020 than in 2019 (83% vs. 73%), and the DKA frequency and HbA1c levels at diagnosis were higher in 2020 than in 2019 (26% vs. 13.4%) [12]. Taken together, these results indicated noticeable effects of SARS-CoV-2 on both the onset and course of diabetes, an effect that should be considered by clinicians.

Our study found an increase in the number of newly diagnosed patients with diabetes during the pandemic (Figure 1), and the rate of COVID-19 in patients with diabetes in Jazan was 38.3%, which was higher compared to other national and local studies conducted during the pandemic (about 26%) (Table 1) [22,23]; furthermore, these results were supported by others [12,19,20,22,23,24]. A literature search was conducted using PubMed with COVID-19 or Coronavirus and T1D as keywords [24]. Trevisani et al. presumed that an increase in diabetes cases since T1D had already been associated with coronavirus respiratory infections, as it happened after the pandemic of SARS-CoV-1 in 2003. Another systematic review and meta-analysis were conducted, reporting that the presence of T1D in patients with COVID-19 ranged from 0.15% to 29%. However, the percentage of COVID-19 in patients with T1D ranged from 0% to 16.67% [25]. Moreover, other studies had similar results, showing an increase in DKA during the COVID-19 pandemic when compared to the previous year [12,19,20,26]. Regarding factors that could be associated with DKA in COVID-19 patients, some studies have shown that individuals with a higher BMI and females are at a higher risk of presenting with DKA, and others observed that a higher BMI and previous pulmonary disease were risk factors for developing long COVID-19 [12,26,27]. However, the results of our study showed that sex and BMI did not affect the development of DKA in patients with COVID-19 (Table 2), and this finding could be limited by the small sample size and absence of information about chronic diseases and long COVID-19.

In contrast to the findings of our study and others (Table 3), a few reports denied the association between SARS-CoV-2 and DKA. For example, a study was conducted in 53 centers in Italy to evaluate whether the diagnosis of T1D or its acute complications changed during the early phase of the COVID-19 pandemic in Italy. The study reported a reduction in new diabetes cases by 23% in 2020 compared to 2019. Of the newly diagnosed patients who presented with DKA in 2020, 44.3% presented with severe manifestations compared to 36.1% in 2019. The study showed no difference in acute complications of diabetes in individuals with COVID-19 before and during the pandemic [28]. Additionally, a study was carried out in Colorado, USA to assess the prevalence of SARS-CoV-2 antibodies in children and adults with and without T1D. They found no difference in the prevalence of SARS-CoV-2 antibodies in children and adults with and without T1D. They found no evidence of a higher COVID-19 rate among young people with newly diagnosed diabetes [29]. Moreover, a study in Poland was conducted by Kucharska et al., where data were collected from the pediatric T1D registry between January 2000 and April 2020. The study revealed that during the months of the COVID-19 pandemic, the prevalence of T1D in children in Lower Silesia was consistent with that of prior years, while the children’s clinical condition was worse during the pandemic than before [30]. These results are inconsistent with our study and suggest that other factors in addition to viral infections could play a significant role in the onset and course of T1D, and further evaluations are warranted to confirm this relationship.

Experimental studies on the DKA and SARS-CoV-2 relationship are limited. DKA could occur as a result of absolute or relative deficits in insulin and an increased counter-regulatory response that results in the production of ketones [31]. ACE2 is a crucial enzyme in the renin-angiotensin-aldosterone system, and it catalyzes the conversion of angiotensin II to angiotensin. ACE2 is set up in the lungs and pancreas and serves as the entry point for SARS-CoV-2 [32]. Once endocytosis of the virus complex occurs, ACE2 expression is downregulated [33]. This allows for the entry of SARS-CoV-2 into pancreatic islet cells, which may generate β-cells’ injury [7]. The downregulation of ACE2 can also lead to unopposed angiotensin II, which may hold back insulin secretion [34]. Moreover, SARS-CoV-2 may aggravate DM by infiltrating and damaging the pancreatic β-cells [35]. In the case of severe COVID-19, insulin resistance may be caused by elevated levels of tumor necrosis factor-alpha (TNFα) and interleukin-6 (IL-6) [36,37]. It was discovered that human pancreatic β-cells and liver organoids were extremely susceptible to SARS-CoV-2 infection [38]. Additionally, SARS-CoV-2 viral antigens were detected in pancreatic β-cells in autopsy samples from patients with COVID-19, and it was reported that many pancreatic islet cells were prone to SARS-CoV-2 [39]. Further, it was found that SARS-CoV-2 infects, and may kill, human pancreatic β-cells in individuals with COVID-19 and that it selectively infects human islet β-cells in vitro [40]. SARS-CoV2 infection can also induce a stress condition in which the activation of the sympathetic nervous system and the hypothalamic–pituitary axis both lead to catecholamine and cortisol production as well as to hyperglycemia, which may potentially increase the risk of T1D onset and DKA development [41]. These factors may have played a part in precipitating DKA and insulin deficiency, a direct cause of newly diagnosed diabetes, as in SARS-CoV-2-infected patients. Another study conducted by Kaya et al. aimed to investigate the presenting characteristics of newly diagnosed T1D patients assessed in the clinic during or before the pandemic. The authors found an increase in the frequency and severity of DKA in children with newly diagnosed T1D in the pandemic period compared to the pre-pandemic period. One factor that could account for this increased incidence of DKA during the pandemic is the restricted utilization of healthcare services due to concern over the spread of SARS-COV-2 [42]. Moreover, some authors presumed that the incidence of DKA could be influenced by changes in blood viscosity following COVID-19 and was less likely associated with an abnormal immune response [43]. In another case series conducted by Singh et al., in which they highlighted that COVID-19 may aggravate DKA in patients with a history of DM, the authors reported eight cases of DKA in COVID-19 patients. The patients were on regular glycemic control medication with uncontrolled HbA1c values that varied from 17–10%. The development of DKA after COVID-19 may be due to uncontrolled DM. However, further studies are needed to address the risk factors for DKA development in COVID-19 patients, with a comparison of DKA incidence among the following groups: undiagnosed DM, diagnosed DM, controlled DM, and uncontrolled DM patients [44].

Another mechanism that should not be ignored is the possible role of insulinopenic hyperglycemia resembling T1D, which is reported to be associated with acute COVID-19 and post-COVID syndrome. Post-COVID syndrome was estimated to affect 10% of patients with variable clinical presentations that tend to persist as a result of multi-organ damage. Though post-COVID syndrome is reported in the older population, 1 to 2% of cases were observed in younger patients. Studies on the immunological background of post-COVID syndrome and its relationship with diabetes are still emerging [45,46].

Despite being one of the few studies in the region that discussed the relationship between COVID-19 and diabetes in a well-established diabetic center and in a region that recorded a high prevalence of diabetes, this study bears many limitations. Due to the nature of this study, there is a potential selection bias. Moreover, during data collection, we needed reported information from patients that was confirmed by contacting patients or their parents. However, this might lead to recall bias and potentially some incorrect information. Furthermore, we failed to follow the time between the diagnosis and the development of DKA in diabetic patients and to include some clinical information, such as a history of chronic diseases, family history, or history of preterm and genetic diseases. Additionally, due to the limitation in our center, we could not perform serological tests that were needed to confirm T1D, and insulin and c-peptide levels were not available at the time of the study.

## 5. Conclusions

Patients newly diagnosed with diabetes during the COVID-19 pandemic are at a higher risk of developing DKA when they are diagnosed with COVID-19, a result that should attract clinicians’ attention to suspected cases of DKA in individuals with previous and newly diagnosed diabetes. Further epidemiological and national studies on a larger population are warranted in order to obtain a better conclusion on the correlation of diabetes and DKA with COVID-19 in a country with a high prevalence rate for diabetes.

## Figures and Tables

**Table 1:** Demographic data of patients included in this study.

Demographic Data of Patients Included in This Study (*n* = 54)			
Variable	Items	*n*	%
Age	Mean	17	-
	Standard deviation	3	-
Sex	Male	23	42.59
	Female	21	57.40
BMI	Underweight	24	44.44
	Normal	23	42.59
	Overweight	6	11.11
	Obese	1	1.85
DKA	Yes	9	16.66
	No	45	83.33
COVID-19	Yes	21	38.88
	No	33	61.11

BMI: Body mass index; DKA: Diabetic ketoacidosis.

**Table 2:** Bivariate analysis according to COVID-19 status.

Variable	COVID-Negative Patients	*n* = 33	COVID-Positive Patients	*n* = 21	95% C.I.	*p*-Value
Age in years Mean:SD	17.15:2.51		16.38:3.66		16.03–17.67	0.464
	*n*	%	*n*	%		
Sex						
Male	18	54.54	13	61.90	0.771–0.788	0.402
Female	15	45.45	8	38.09		
BMI						
Underweight	13	39.39	11	52.38	0.355–0.374	0.714
Normal	15	45.45	8	38.09		
Overweight	4	12.12	2	09.52		
Obese	1	03.03	0	0		
DKA						
Yes	2	06.06	7	33.33	0.016–0.021	0.020 *
No	31	93.93	14	66.66		

SD: Standard deviation. C.I.: confidence interval. BMI: Body mass index. DKA: Diabetic ketoacidosis. * The alpha criterion for the *p*-value was set to 0.05.

**Table 3:** Studies reported DKA among patients with COVID-19.

Country [Reference]	Year of Study	Study Population	Reported New-Onset Diabetic Patients with DKA and COVID-19
Saudi Arabia [12]	2020	260	26%
UK [18]	2020	30	47%
Germany [19]	2020	532	45%
Israel [20]	2020	146	58%
UK [21]	2020	33	52%
Current study	2020	54	33%

## Data Availability

Data are available upon request from the corresponding author.
